# Comparison of hepatitis B, hepatitis C, and HIV seropositivity of Syrian and Turkish pregnant women

**DOI:** 10.4274/tjod.galenos.2019.15564

**Published:** 2019-07-03

**Authors:** Pınar Yalçın Bahat, Gökçe Turan, Bahar Yüksel Özgör, Kübra Bağcı Çakmak

**Affiliations:** 1 Universit of Health Sciences, Kanuni Sultan Süleyman Training and Research Hospital, Clinic of Obstetrics and Gynecology, İstanbul, Turkey; 2 Kırıkhan State Hospital, Clinic of Obstetrics and Gynecology, Hatay, Turkey; 3 Esenler Obstetrics and Pediatrics Hospital, Clinic of Obstetrics and Gynecology, İstanbul, Turkey

**Keywords:** Hepatitis B, hepatitis C, HIV, pregnancy, seroprevalence

## Abstract

**Objective:**

In this study, we aimed to compare the seroprevalence of hepatitis B (HBV), hepatitis C (HCV), and human immunodeficieney viruse (HIV) in Syrian pregnant women and Turkish pregnant women in our hospital.

**Materials and Methods:**

In our study, a total of 68,169 Turkish pregnant women who received HB surface antigen (HBsAg), HB surface antibody (anti-HBs), HCV antibody (anti-HCV), anti-HIV test, and a total of 11,015 Syrian pregnant patients who received HBsAg, anti-HBs, anti-HCV and anti-HIV tests were examined retrospectively between January 2012 and January-2018 in Universty of Health Sciences Kanuni Sultan Süleyman Training and Research Hospital, Obstetrics and Gynecology Clinic.

**Results:**

In our study, the rates of HBsAg, anti-HCV, and anti-HIV seropositivity were 1.1%, 0.1%, and 0.03% in Syrian pregnant women between 2012 and 2018, respectively. In the other study group, in Turkish pregnant women, HBsAg, anti-HCV, and anti-HIV seropositivity rates for 2012 and 2018 were found as 1.8%, 0.2%, and 0.08%, respectively.

**Conclusion:**

Although there were no significant differences between the HBsAg, anti-HCV, and anti-HIV results of both groups, the anti-HBs positivity was higher at a significant level in Turkish pregnant women. The reason of the significantly higher anti-HBs positivity levels in pregnant women might stem from the fact that women are vaccinated and controlled regularly due to the policies in this regard in our country.

**PRECIS:** This study was conducted to compare the seroprevalence of hepatitis B, Hepatitis C, and HIV in Syrian pregnant women and Turkish pregnant women.

## Introduction

Infection is one of the most important factors increasing perinatal morbidity and mortality. Studies have shown that infections that present during the gestational period have the risk of infecting the fetus by exceeding the placenta and increase fetal mortality and morbidity^(1)^.

Since 2011, because of the civil war, about 2.5 million Syrian people have been forced to abandon their countries and live in refugee camps in neighboring countries. Syrians have been provided with temporary protection by Turkey and are the densest group of asylum seekers in our country. Approximately 2.7 million Syrian refugees have already been distributed in Turkey, which has world’s largest population of Syrian refugees^(2)^. Refugees may face housing, food, medical accessibility, and language barriers when they come to temporary or new host countries. The Turkish Government has provided free healthcare for Syrian refugees, and the facilities to health services has been increased.

The rates of pregnancy and birth are high in Syrian refugees in our country^(3)^. Due to limited opportunities in communication, healthcare workers are also affected and difficulties are experienced in health services. For these reasons, adequate measures against infectious diseases cannot be taken and the mother, fetus, and health workers are at risk. The failure of Syrian pregnant women to adapt to Turkish screening and vaccination programs, and most Syrian pregnant women being seen by physicians during the first birth is a common problem.

This study was conducted to compare the hepatitis B virus (HBV), hepatitis C virus (HCV), and human immmunodeficiency virus (HIV) seropositivity of Turkish pregnant women and that of Syrian migrant pregnant women who gave birth in our hospital.

## Materials and Methods

Our study was performed retrospectively after approval was obtained from the Local Ethics Committee of University of Health Sciences Kanuni Sultan Suleyman Training and Research Hospital (approval number: 2018.10.36). A total of 11,015 Syrian pregnant women and 68,169 Turkish pregnant women who presented due to pregnancy and who gave birth were included in the study. The women presented to University of Health Sciences Kanuni Sultan Süleyman Training and Research Hospital, Clinic of Obstetrics and Gynecology of İstanbul University of Health Sciences between 2012 and 2018. Patients’ files were retrospectively scanned and their ages and ethnicity (Syrian refugee-Turkish population) were recorded. Venous blood samples from all patients were tested for HBsAg, anti-HBs, anti-HCV and anti-HIV using the micro-ELISA method. Suspected positive anti-HIV sera samples were confirmed using the western blot method.

### Statistical Analysis

The Statistical Program for the Social Sciences (SPSS Chicago, IL, USA) program was used to evaluate all collected data. Continuous variables with normal distribution were reported as the average. P values less than 0.05 were considered statistically significant.

## Results

In the study, 11,015 Syrian immigrant pregnant women and 68,169 Turkish pregnant women were compared in terms of serology. The serology results of the study and control groups are given in Table 1. A total of 68,169 Turkish patients who gave birth in our hospital and 11.015 Syrian patients were examined for HBsAg, 67,760 Turkish and 11,004 Syrian pregnant for anti-HCV, 67,871 Turkish and 11,015 Syrian pregnant women for anti-HIV, and 7130 Turkish and 180 Syrian pregnant women for anti-HBs. The average age of the Turkish women (28±6 years) was significantly higher than that of the Syrian migrant women (25±6.02 years) (p<0.001).

Anti-HCV was positive in 0.2% of 67,760 Turkish pregnant women and 0.1% of 11,004 Syrian pregnant women. There was no statistically significant difference between anti-HCV positivity of either group.

Anti-HIV was positive in 57 of 68,169 Turkish pregnant women, 12 of these patients were confirmed and seen as negative in our records. The other patients’ verification results could not be obtained. Anti-HIV was positive in 4 cases of 11,015 Syrian pregnant women, and four of our patients were found to be negative in our records of verification.

When HBsAg positivity of both groups was examined, HBsAg was studied in all pregnant women who gave birth in our hospital. HBsAg positivity was found in 1.8% of the 68,169 Turkish pregnant women and in 1.1% of the 11,015 Syrian pregnant women.

Although the total number of patients studied for anti-HBs was less than the others, 26.3% of 7130 patients with anti-HBs in Turkish pregnant women were positive, and in Syrian patients, only 180 patients were examined and 11% of them were positive. When these two groups were compared with each other, anti-HB outcomes were significantly higher in Turkish patients.

Syrian pregnant women were divided into two groups, as those who were under and over the age 35 years. When the serologic results were compared between the two groups, both anti-HCV and HBsAg were found as significantly higher for patients over the age of 35 years (p<0.001 and p=0.002, respectively). There was no statistically significant difference of anti-HIV and anti-HB positivity between the two groups. The serologic results of these groups are given in Table 2.

## Discussion

Since 2011, Syrians have migrated to many countries due to the Syrian civil war between rebels and government forces. The majority of the refugees have chosen to be refugees in Turkey. According to the April 2016 United Nations Refugee Agency data, there were 2,749,140 registered Syrian refugees in Turkey^(2)^. The increasing number of uncontrolled and informal Syrian refugees causes many social and health problems. It is important to know the hepatitis and HIV prevalence in both the refugee and the local community.

There is no congenital anomaly or teratogenic effect of HBV and it does not pass through the placenta^(4)^. When the case of infection from the mother to the infant is considered, it may be due to contact with various maternal fluids during or after childbirth or in the vagina during vaginal delivery, or by swallowing mother’s blood or after a placental injury^(5)^. HBsAg has been shown in the mother’s milk, and theoretically, it can be considered as a breast milk infectious agent; however, infectiousness is decreased by 85-95% with the newborn vaccination program, which is routine in our country^(6)^.

HBV infection is an important health problem in our country as it is in developing countries. In recent years, the screening of donors for hepatitis infection, progress in the sterilization of instruments used in healthcare, and the increase of disposable materials has reduced the infectivity of the infection to a relatively small extent. In addition, HBV vaccination has been routinely practiced in our country since 1998 at the 0 years age group. Considering that HBV vaccine administered in newborns and immunoglobulins significantly prevent vertical passage, it shows us how important it is for pregnant women to be screened for HBsAg.

HCV infection may also pass vertically through the newborn. Babies of high-viral-loaded mothers are at greater risk. However, this ratio is lower than that of HBV^(7)^. It has been observed that most HIV infections are seen in childhood in the perinatal period and this transition is between 13-43%. It is known that infants of pregnant women who are known to be HIV-positive and who are treated with zidovudine during their pregnancies have a 25% reduction in the risk of transmission from mother to infant with ongoing postpartum 6-week zidovudine treatment^(8)^.

There are many studies in the literature showing the maternal outcomes of refugees from different ethnic groups, but there are few studies comparing HBV, HCV, and HIV infections of the Syrian population in Turkey. In the study conducted by İnci et al.,^(9)^ on 4186 pregnant women including 2158 Syrians and 2028 Turks, the rate of vaccination of pregnant women was investigated, and the HBsAg positivity rates before and after vaccination were analyzed. HBsAg positivity was 1.4% among all pregnancies, which was 1.8% among Turkish women and 1.1% among Syrian women.

In the study of Çift et al.,^(10)^ comprising 297 Syrian refugees and 300 Turkish women, a total of 597 participants who presented due to pregnancy and giving birth, the anti-HBs immunoglobulin G (IgG) positivity ratio in Turkey (13.9%) was found to be statistically higher than in Syria (8.5%). Again, in this study, the rates of HBsAg and anti-HCV in Syrian pregnancies were found as 0.3%, and they were 0.8% and 0%, respectively, in Turkish pregnant women. In our study, HBsAg and anti-HCV positivity were found as 1.8% and 0.2% in Turkish women and 1.1% and 0.1% in Syrian pregnant women, respectively. However, the difference between this study and our study is that there were no anti-HBs IgG and IgM data of the patients.

In the study conducted by Madendag et al., ^(11)^ which only investigated Turkish pregnant women, 1,910 HBsAg positivity of 90.351 pregnancies (2.11%), anti-HCV positivity in 102 of 60,729 pregnant women (0.17%), and anti-HIV positivity was detected in 3 of 60,562 pregnancies (0.004%).

In a study conducted by Coppola et al.^(12)^ on HBV infection on 1212 immigrants in Italy, they found HBsAg positivity in 116 patients (9.6%). A total of 606 (50%) patients had negative HBsAg/anti-HB antibody values. It has been reported that immigrants have to undergo HBV vaccination after 4.5 years of living in Italy because immigrants’ serology has not been assessed, no vaccination has been performed, and immunization has not been provided so far.

Considering the number of Syrian pregnancies in our country and a group of patients who were not previously on the screening program, HBV, HCV, and HIV screening of patients is very important both for newborn health and for the safety of our healthcare. Vaccination against hepatitis B is possible and immunity is mandatory for both Syrians and people who were born before 1998 and who are not on the vaccination schedule. There is no vaccine or prophylactic treatment developed for HCV and HIV, and therefore, all pregnancies, especially among Syrian pregnancies, should be routinely screened, materials such as gloves, masks, and glasses should be used during the examination or surgery and the risk of infection should be reduced^(10)^.

The authors also reported maternal and infant infections (including HIV, toxoplasmosis, sexually transmitted infections and rubella seronegativity) to be worse amongst migrant women in 63.6% of included studies and better in 9.1%; the remaining studies showed mixed results and reported that admission to an neonatal intensive care unit (NICU) or special care was higher amongst offspring of migrant women^(13)^.

In the European Union, a number of communicable diseases have been reported to spread in the refugee population including acute respiratory tract infections, louse-borne relapsing fever, cutaneous diphtheria, scabies, measles, meningococcal meningitis, shigellosis, typhoid fever, hepatitis A (HAV), tuberculosis, and malaria. Across studies, tuberculosis - particularly latent - and HBV are the most commonly reported diseases. A recent study including only Syrian refugees found leishmaniasis, tuberculosis, hepatitis, and vitamin D insufficiency to be the most prevalent health concerns^(14)^. A study from Italy of 529 asylum seekers found 8.3% to be HBsAg positive and 45.6% to be anti-HBV positive^(15)^.

The inclusion of Syrian pregnant women and their newborns in our country in the national vaccination and screening program, in addition to providing them with treatment, should become the priority health policy. According to our study, it is suggested that hepatitis and HIV screening should be performed in medical treatments because Syrian refugees migrate from regions where the disease is prevalent.

### Study Limitations

The limitation of the present study was its retrospective nature, and therefore, the anti-HBs IgG and IgM results of the patients could not be obtained. However, the strong side of the present study is that the number of the cases was higher and it provided insight on seropositivity comparison.

## Conclusion

As a result, although there were no differences between HBsAg, anti-HCV, and anti-HIV results in our study, anti-HB positivity was found to be higher at a significant level in Turkish pregnant women. The reason why the anti-HB positivity scores were higher in Turkish pregnant women might be because of the regular and controlled vaccination policies in our country. We believe that it is important that the routine screening and awareness programs are organized on the prevalence of HBV, HCV, and HIV among both Turkish pregnant women and in refugees due to the increasing Syrian population living in our country. We also believe that planning new treatments such as immunization, immunoglobulin or zidovudine is a preventive and therapeutic method. Thus, it will be possible to reduce postnatal mortality and morbidity due to infection. In addition, healthcare staff who are at risk will be safeguarded in this way in terms of infectious diseases.

## Figures and Tables

**Table 1:**
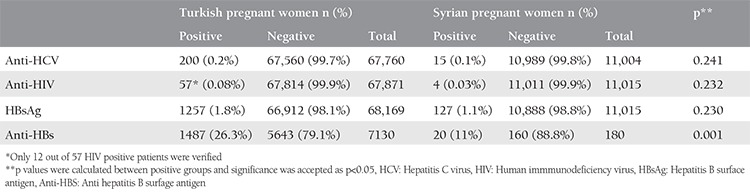
Comparison of serology results of Turkish and Syrian immigrant pregnant women

**Table 2:**
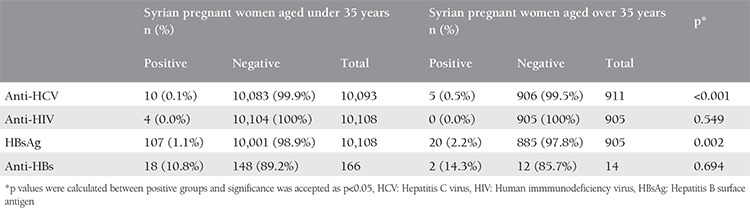
The analysis of Syrian pregnant women based on age
